# ROS Reduction Does Not Decrease the Anticancer Efficacy of X-Ray in Two Breast Cancer Cell Lines

**DOI:** 10.1155/2019/3782074

**Published:** 2019-03-14

**Authors:** Huizhen Wang, Xin Zhang

**Affiliations:** ^1^High Magnetic Field Laboratory, Key Laboratory of High Magnetic Field and Ion Beam Physical Biology, Hefei Institutes of Physical Science, Chinese Academy of Sciences, Hefei, Anhui 230031, China; ^2^University of Science and Technology of China, Hefei, Anhui 230036, China; ^3^Institute of Physical Science and Information Technology, Anhui University, Hefei, Anhui 230601, China

## Abstract

Radiotherapy is effective on a large number of cancer types and is one of the most frequently administrated treatments for cancer patients. The anticancer efficacy of X-ray radiotherapy has been frequently correlated with reactive oxygen species (ROS) elevation, which is also a limiting factor for its toxicity on normal tissues. Here, we found that although 4-10 Gy X-rays could significantly reduce cell numbers in both MDA-MB-231 and MCF-7 breast cancer cells, the ROS level changes are less in MCF-7 cells than in MDA-MB-231 cells. Moreover, although both the ROS scavenger N-acetyl-L-cysteine (NAC) and 1 T static magnetic field (SMF) could reduce X-ray-induced ROS elevation, they did not prevent X-ray-induced cell number reduction or cell death increase, which is significantly different from cisplatin. These results demonstrate that although the anticancer efficacy of cisplatin on two breast cancer cell lines is dependent on ROS, the anticancer efficacy of X-ray is not. Moreover, by testing 19 different cell lines, we found that 1 T SMF could effectively reduce ROS levels in multiple cell lines by 10-20%, which encourages further studies to investigate whether SMF could be used as a potential “physical antioxidant” in the future.

## 1. Introduction

Radiotherapy has great advantages over chemotherapy for generating localized ionizing radiation on tumor tissues while fewer effects on normal tissues in the human body. Overall, radiotherapy is currently estimated to be used on around 50% of cancer patients and contributes to about 40% of curative treatment for cancers [1, 2].

Although different cell types and tissues respond to radiation differentially [3–5], the anticancer efficacy of X-ray radiotherapy has been frequently correlated with increased reactive oxygen species (ROS) and apoptosis [6–12]. Theoretically, precisely positioned high-energy X-ray or *γ*-ray could kill cancer cells by directly or indirectly damaging DNA structure or by overproducing ROS in cells to break down DNA [13, 14]. ROS are highly reactive oxygen metabolites that include multiple types, such as superoxide radical, hydrogen peroxide, and hydroxyl radical. They are normal metabolic products during cell respiration, which are essential for some physiological cellular processes, but can be elevated in some pathological conditions or under external stresses.

There are multiple evidences showing that cancer cells generally have higher ROS levels than noncancer cells [15, 16]. Since excessive ROS could cause DNA damage, attack various macromolecules, damage cellular components and accumulate oxidative damage, they are considered to be a possible cause for carcinogenesis. On the other hand, elevated ROS could also be used as an effective way to kill cancer cells for their apoptosis-promoting ability [17, 18]. Moreover, it is proposed that cancer cells may be more sensitive than normal cells to ROS accumulation so that increased oxidative stress by exogenous ROS generation could be used as an anticancer therapy strategy to selectively kill cancer cells without affecting normal cells [19–21]. Therefore, ROS are considered to be a double-edged sword in tumorigenesis and cancer treatment.

Although radiation is much more localized on targeted cancer tissues than chemotherapy drugs, it could also cause side effects on other tissues. Considering the prevalence of ROS elevation after radiation, people have used antioxidant agents not only to reduce radiation-induced apoptosis, tissue damage, and improve rodent survival in researches [22–27] but also as radioprotective agents to alleviate side effects on patients [28, 29]. However, there are some studies showing that supplementation with high doses of antioxidant alpha-tocopherol and beta-carotene during radiotherapy might compromise radiotherapy efficacy in head and neck cancers [30, 31], which could be caused by decreased ROS levels. But whether reducing ROS will reduce radiotherapy efficacy on other cancer types is still unknown.

Here in this study, we investigated the dependency of ROS in the anticancer efficacy of X-ray on breast cancer cells. Since breast cancer is a complex disease that has different subtypes based on gene expression and histological signatures [32, 33], we chose both the luminal breast cancer cell line MCF-7 and the mammary gland breast cancer cell line MDA-MB-231. We found that the anticancer efficacy of cisplatin in these breast cancer cells is ROS dependent, but the anticancer efficacy of X-ray is ROS independent. Moreover, the results from 19 different cell lines show that 1 T SMF could reduce ROS levels in multiple cell types.

## 2. Materials and Methods

### 2.1. Cell Culture

We tested 19 cell lines in this study, which are all adherent cells. The GIST-T1 cell line (gastrointestinal stromal tumor) was from Cosmo Bio Co. Ltd. (Tokyo, Japan). All other cell lines were from American Type Culture Collection (Manassas, VA, USA).

MCF-7, MDA-MB-231, GIST-T1, HeLa (cervix epithelial adenocarcinoma), HCT116 (colon epithelial carcinoma), PC3 (prostate adenocarcinoma), C6 (rat brain glial), HepG2 (hepatocellular carcinoma), RPE1 (retina epithelial), 293T (kidney epithelial), differentiated PC-12 (rat pheochromocytoma), NIH-3T3 (mouse embryo fibroblast), and CHO (Chinese hamster ovary) cells were cultured in DMEM medium without L-glutamine (15-017-CVR, Corning, NY, USA), supplemented with 10% (*v*/*v*) FBS (fetal bovine serum) (FB25015, Clark Bioscience, Richmond, VA, USA), 1% GlutaMAX (35050-061, Gibco, Carlsbad, CA, USA), and 1% (*v*/*v*) P/S (penicillin/streptomycin) (SV30010, HyClone, Logan, UT, USA). CNE-2Z (nasopharyngeal cancer), EJ1 (bladder cancer), and U251 (brain glioblastoma) cells were cultured in RPMI 1640 without L-glutamine (15-040-CVR, Corning) and supplemented with 10% FBS, 2 mM GlutaMAX, and 1% P/S. Three human noncancer lung cells (HSAEC2-KT, HSAEC30-KT, and HBEC30-KT) were cultured in SAGM (Lonza, CC-3118). All cells were maintained in a cell incubator (BC-J160S, Shanghai Boxun, Shanghai, China) at 37.0°C and 5% CO_2_.

### 2.2. X-Ray Irradiation

The MCF-7 and MDA-MB-231 cells were plated at 4 × 10^5^/ml one day before the experiment to allow cell attachment. On the second day, cells were irradiated with a cabinet X-ray machine (X-RAD 320, Precision X-Ray Inc., North Branford, CT, USA) at 12.5 mA and 320 kV, using a dose rate of 1.04 Gy/min, to a total dose of 4/6/8/10 Gy or 4/8 Gy in specific experiments, and then incubated in a humidified atmosphere at 37.0°C and 5% CO_2_ for another 2 days, with or without SMF exposure. Finally, the samples were harvested to measure cell number, ROS level, cell death, cell cycle, and clonogenic survival assay.

### 2.3. Static Magnetic Field Exposure

1 T SMF was provided by permanent magnets and the details have been described in our previous studies [34, 35]. We used the north pole of the magnet for cell exposure in this study and the magnetic field intensities at the position of the cells were 1.07 ± 0.037 T. To minimize the experimental variations, the control groups were placed in the same cell incubator, but far away from the magnets where the magnetic field intensity was 0.925 ± 0.206 Gs (background magnetic field in the lab was 0.875 ± 0.171 Gs), which is 10,000-fold lower than the 1 T SMF experimental group.

### 2.4. NAC and Cisplatin Treatment

N-Acetyl-L-cysteine (NAC, acetylcysteine, HY-B0215) and cisplatin (HY-17394) were purchased from MedChemExpress (Shanghai, China). The NAC stock solution was made by dissolving NAC in deionized H_2_O at 200 mM, adjusting the pH to 7.2-7.4 before it was filtered with 0.22 *μ*m filters. The cisplatin stock solution was made at 10 mM in N, N-dimethylformamide (DMF), which was used as control for cisplatin addition. Cells in the NAC treatment groups (NAC, NAC + 1 T SMF,NAC + 4/8 Gy, NAC + cisplatin, and NAC + cisplatin + 8 Gy) were preincubated with 10 mM NAC for 1 h. Then, the cells were treated with specific conditions according to experimental designs and maintained in a cell incubator at 37.0°C and 5% CO_2_, with or without 1 T SMF exposure for another 2 days before they were harvested for analysis.

### 2.5. Measurement of Intracellular ROS Levels

Intracellular ROS levels were detected using the fluorogenic probe 2′, 7′-dichlorofluorescin diacetate (DCFH-DA, D6883, Sigma-Aldrich, St. Louis, MO, USA), which is a cell-permeable nonfluorescent probe. DCFH-DA can easily pass the cell membrane and is deesterified by intracellular esterases to the nonfluorescent polar derivative DCFH, which is oxidized to highly fluorescent dichlorofluorescein (DCF) in the presence of ROS.

DCFH-DA was used as previously described in the literature [36–38]. Basically, cells were harvested and loaded with 2 *μ*M DCFH-DA at 37°C for 20 min in DMEM medium without serum. The working volume DCFH-DA was added proportionately, which was based on the total cell numbers. After PBS washes, the DCF fluorescence in the cells was measured by a flow cytometer (CytoFLEX, Beckman Coulter, Brea, CA, USA) at an excitation wavelength of 500 nm. For each sample, 10,000 events were acquired and the raw data of geometric mean of fluorescence were quantitatively analyzed using the CytExpert Software program (version 1.2). Relative fluorescence was calculated by normalization to the control group.

We also used carboxy-H_2_DCFDA (C400, Molecular Probes, Eugene, OR, USA) probe, which is oxidation insensitive, to measure intracellular ROS level. As a positive control, H_2_O_2_ was incubated with cells for 30 min. We used different H_2_O_2_ concentrations because various cell types responded differently. After preliminary examination, we chose 400 *μ*M for MDA-MB-231, 2 mM for MCF-7, 1 mM for 293T, and 3 mM for RPE1 cells. H_2_O_2_ at these concentrations could increase the ROS levels in these cells without causing dramatic cell death. Cells with or without 1 T SMF exposure were collected, and then incubated with 25 *μ*M carboxy-H_2_DCFDA at 37°C for 1 h in DMEM medium without serum. After being washed by PBS, 10,000 cells were measured by flow cytometer at an excitation wavelength of 495 nm. Relative fluorescence intensity of 1 T SMF treatment groups was normalized to the control groups.

### 2.6. Clonogenic Survival Assay

The cells were plated one day ahead and then pretreated with or without 10 mM NAC for 1 hour before DMF vehicle control or cisplatin 1/2/5 *μ*M addition. Then, they were cultured for another two days in the presence or absence of 1 T SMF. The X-ray in combination with NAC/1 T SMF experimental procedures is the same as described above. Then, the cells were harvested, counted, and resuspended in fresh complete DMEM. 500 cells were plated per well and maintained in a cell incubator for 12-14 days, with medium change every 3 days. At the end of the experiment, cells were fixed by 4% formaldehyde for 20 min, stained with 0.1% crystal violet solution for another 20 min, followed by PBS wash for several times. Colonies with more than 50 cells were counted. The efficiency of colony formation was calculated as [(colonies counted/cells seeded) × 100] %. All experiments were conducted in triplicate and repeated at least three times.

### 2.7. Cell Death Analysis

We used FITC Annexin V Apoptosis Detection Kit (556547, BD Pharmingen™, San Diego, CA, USA) for cell death analysis according to the manufacturer's instructions. Briefly, cells were harvested and washed with PBS before they were resuspended in 100 *μ*l binding buffer. Then, 5 *μ*l FITC Annexin V and 10 *μ*l PI (propidium iodide) were added to the cell suspensions, mixed, and incubated in the dark for 20 min at room temperature. Then, 400 *μ*l binding buffer was readded to the cells before they were analyzed by flow cytometry. 10,000 events were collected and analyzed for each sample. Experiments were conducted at least three times independently. Both apoptotic and necrotic cells were analyzed, and their numbers were combined, normalized to the control group, and shown as “relative dead cell number” in the figures.

### 2.8. Cell Cycle Distribution Analysis

Cells were trypsinized and washed with PBS before they were fixed by 70% ice-cold ethanol overnight at -20°C. Then, the cells were rewashed by PBS and incubated in PI solution (BD Pharmingen, San Diego, CA, USA) for 30 min at room temperature in the dark. Samples were collected using flow cytometry and data were analyzed by ModFit LT.

### 2.9. Statistical Analysis

In the current manuscript, all experiments were repeated at least three times independently, and the data were analyzed by GraphPad Prism 5 (version 5.01, GraphPad Software, La Jolla, CA, USA). Mean values are shown in the figures, and SDs are shown as error bars. Comparisons between treatments were analyzed by a two-tailed Student's *t*-test. *p* values are labeled in the figures for where data were compared or between the experimental group and its control group.

## 3. Results

**Figure 1:**
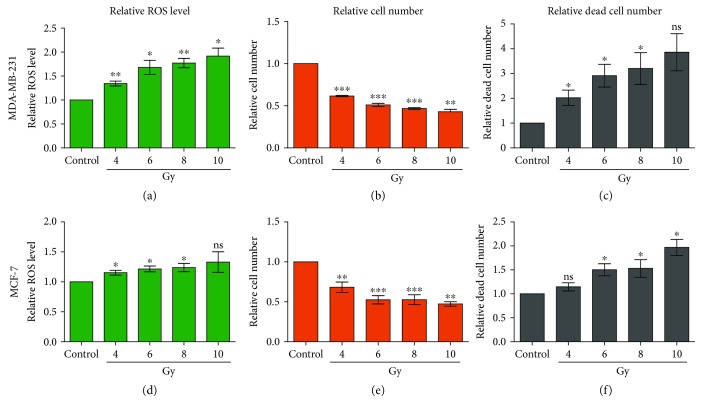
X-rays significantly increase the intracellular ROS level and cell death and decrease cell numbers in MDA-MB-231 and MCF-7 cells. The relative ROS level (a, d), relative cell number (b, e), and relative dead cell number (c, f) were measured in MDA-MB-231 and MCF-7 cells 48 hours after 4/6/8/10 Gy X-ray irradiation. ^∗^ *p* < 0.05, ^∗∗^ *p* < 0.01, ^∗∗∗^ *p* < 0.001; ns: not significant.

We first examined the effects of 4/6/8/10 Gy X-rays on MDA-MB-231 breast cancer cells. As expected, the ROS levels were significantly increased by X-rays at all doses (Figure 1(a)). The cell numbers were reduced, and cell death was increased in a dose-dependent way (Figures 1(b) and 1(c)). However, MCF-7 breast cancer cells responded to X-rays similarly but to a less extent. The ROS levels in MCF-7 cells were increased by <20% after 4-10 Gy X-ray treatment (Figure 1(d)), which is much lower than the 40-90% in MDA-MB-231 cells (Figure 1(a)). However, the MCF-7 cell numbers were reduced markedly, and cell death was also increased (Figures 1(e) and 1(f)), which is similar to MDA-MB-231 cells.

**Figure 2:**
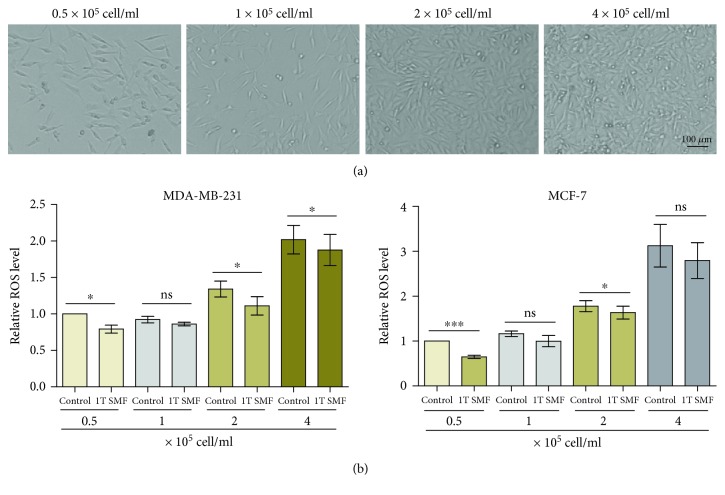
1 T static magnetic field decreases the intracellular ROS level in both MCF-7 and MDA-MB-231 cells at different cell densities. Cells were plated at 0.5/1/2/4 × 10^5^/ml and treated with 1 T SMF for one day. Bright field images were taken before they were harvested and measured for ROS levels. Comparisons were made between the experimental group and the control group using a Student's *t*-test. ^∗^ *p* < 0.05, ^∗∗∗^ *p* < 0.001; ns: not significant.

It has been previously reported that the ROS levels can be affected by many factors, such as cell density and magnetic fields of various types [39, 40]. We found that for both MDA-MB-231 and MCF-7 cells, the ROS levels were significantly elevated when the cell plating densities were increased, which means that these breast cancer cells generate higher levels of ROS when they are more crowded (Figure 2(a)). It is obvious that 1 T static magnetic field (SMF), with the north pole beneath the cells (Supplementary Figure 1), can reduce the ROS level in both cell lines at multiple cell densities (Figure 2(b)).

**Figure 3:**
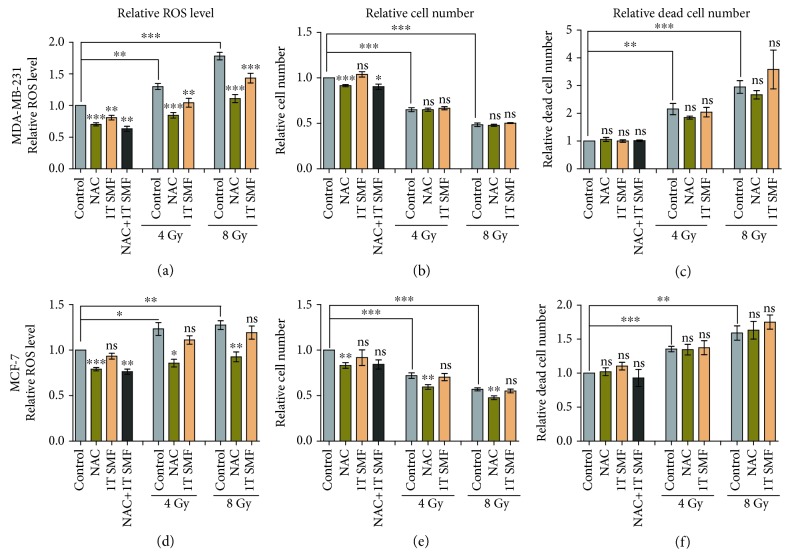
The anticancer effects of X-rays on MDA-MB-231 and MCF-7 cells were not reversed by the ROS scavenger NAC or 1 T static magnetic field. The relative cellular ROS level (a, d), relative cell number (b, e), and relative dead cell number (c, f) were measured for MDA-MB-231 and MCF-7 cells treated with or without NAC, 1 T SMF, and X-rays. Comparisons were made between the experimental group and the control group using a Student's *t*-test. ^∗^ *p* < 0.05, ^∗∗^ *p* < 0.01, ^∗∗∗^ *p* < 0.001; ns: not significant.

Next, we used both NAC and 1 T SMF to test the dependence of X-ray-induced breast cancer cell reduction on ROS. NAC is a total ROS scavenger that can react with various ROS, including hydrogen peroxide, hydroxyl radical, superoxide, and hypochlorous acid, which has been used to treat multiple diseases such as chronic obstructive pulmonary disease (COPD) and acetaminophen overdose [41–46]. It is surprising that although both NAC and 1 T SMF could reduce cellular ROS significantly in control and X-ray-radiated MDA-MB-231 cells (Figure 3(a)), the X-ray-induced cell number reduction and cell death increase were not prevented (Figures 3(b) and 3(c)). Similarly, in MCF-7 cells, the anticancer effects of X-rays were not reversed by NAC or 1 T SMF either (Figures 3(d)–3(f)). On the contrary, NAC can even potentiate the antitumor effects of 4/8 Gy X-rays on cell number (Figure 3(e)). These results further prove that X-ray reduces these two types of breast cancer cell numbers in an ROS-independent way.

**Figure 4:**
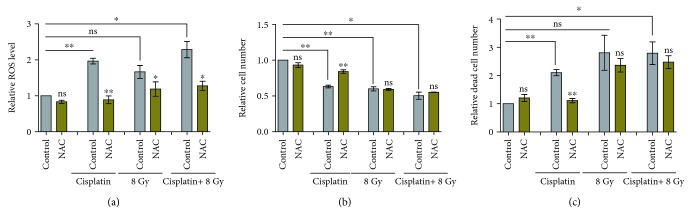
The effects of cisplatin on MDA-MB-231 ROS level, cell number, and cell death are ROS dependent. The relative ROS level (a), cell number (b), and dead cell number (c) were measured for MDA-MB-231 cell treated with 2 *μ*M cisplatin and/or 8 Gy X-ray, in the presence or absence of NAC. Comparisons were made between the experimental group and the control group using a Student's *t*-test. ^∗^ *p* < 0.05, ^∗∗^ *p* < 0.01, ^∗∗∗^ *p* < 0.001; ns: not significant.

Next, we compared the effects of X-ray on MDA-MB-231 cells to that of cisplatin, a chemotherapy drug that was reported to increase the cellular ROS levels. For both cisplatin and X-ray, the ROS level in MDA-MB-231 could be significantly increased (around 2-fold of control), which could be effectively prevented by adding NAC (Figure 4(a)). It is obvious that NAC could only significantly reverse cisplatin-induced cell number reduction and cell death increase but not that of 8 Gy X-ray (Figures 4(b) and 4(c)). These results demonstrate that ROS are indispensable for breast cancer cell number reduction and cell death increase induced by cisplatin but not X-ray radiation.

**Figure 5:**
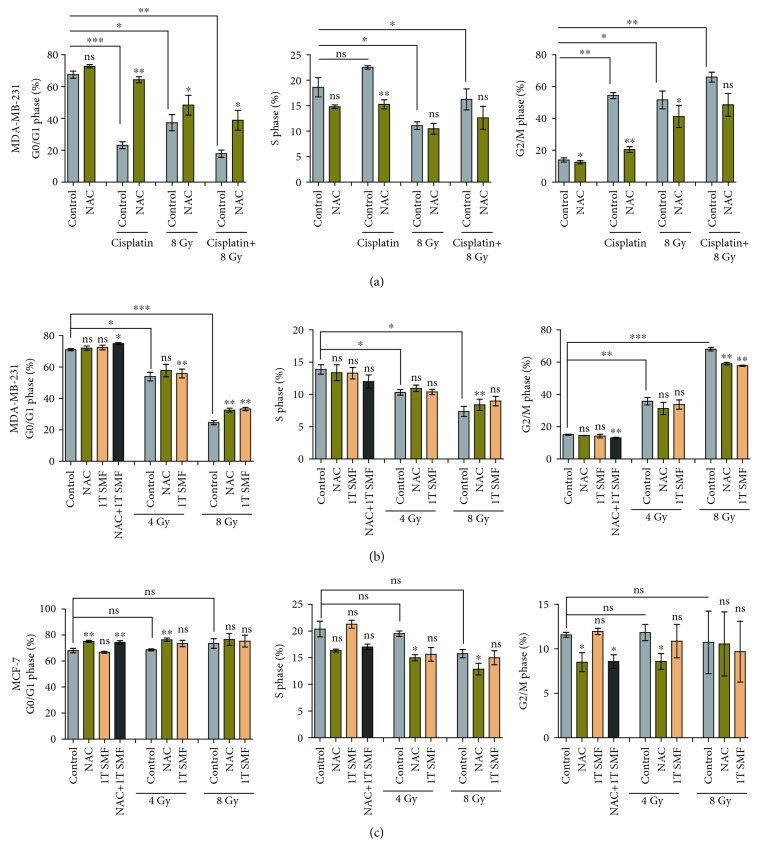
ROS reduction abolished cisplatin-induced cell cycle arrest but only moderately reversed X-ray induced cell cycle arrest. (a) The cell cycle distribution was measured for MDA-MB-231 cell treated with 2 *μ*M cisplatin and/or 8 Gy X-ray, in the presence or absence of NAC. MDA-MB-231 cells (b) and MCF-7 cells (c) were treated with or without NAC, 1 T static magnetic field, and X-rays before their cell cycle were measured. Comparisons were made between the experimental group and the control group using a Student's *t*-test. ^∗^ *p* < 0.05, ^∗∗^ *p* < 0.01, ^∗∗∗^ *p* < 0.001; ns: not significant.

Since cell number reduction can be caused not only by cell death but also by cell cycle alterations, we examined the cell cycle distribution treated with X-rays or cisplatin, with or without ROS scavenging (Figure 5). It is obvious that NAC completely abolished the cell cycle arrest caused by cisplatin but only moderately reversed the cell cycle perturbation effects of 8 Gy X-ray in MDA-MB-231 cells (Figure 5(a)), which indicates that ROS is essential for cisplatin but not 8 Gy X-ray-induced cell cycle perturbation. We then further analyzed the cell cycle distribution of both MDA-MB-231 and MCF-7 cells for 4 Gy and 8 Gy X-rays with or without ROS reduction by NAC or 1 T SMF (Figures 5(b) and 5(c)). The raw data of cell cycle analyses were shown in Supplementary Figure 2. It is interesting that X-ray induced significant cell cycle arrest in MDA-MB-231 cells (Figure 5(b)), but not in MCF-7 cells (Figure 5(c)), which is probably related to the less extent of ROS elevation caused by X-rays in MCF-7 (Figures 1 and 3). Moreover, although X-ray-induced cell cycle arrest was only moderately inhibited by NAC and 1 T SMF, which is not as dramatic as the cisplatin-induced cell cycle arrest, these results indicate that ROS elevation at least partially contributed to the X-ray-induced cell cycle arrest in MDA-MB-231 breast cancer cells.

**Figure 6:**
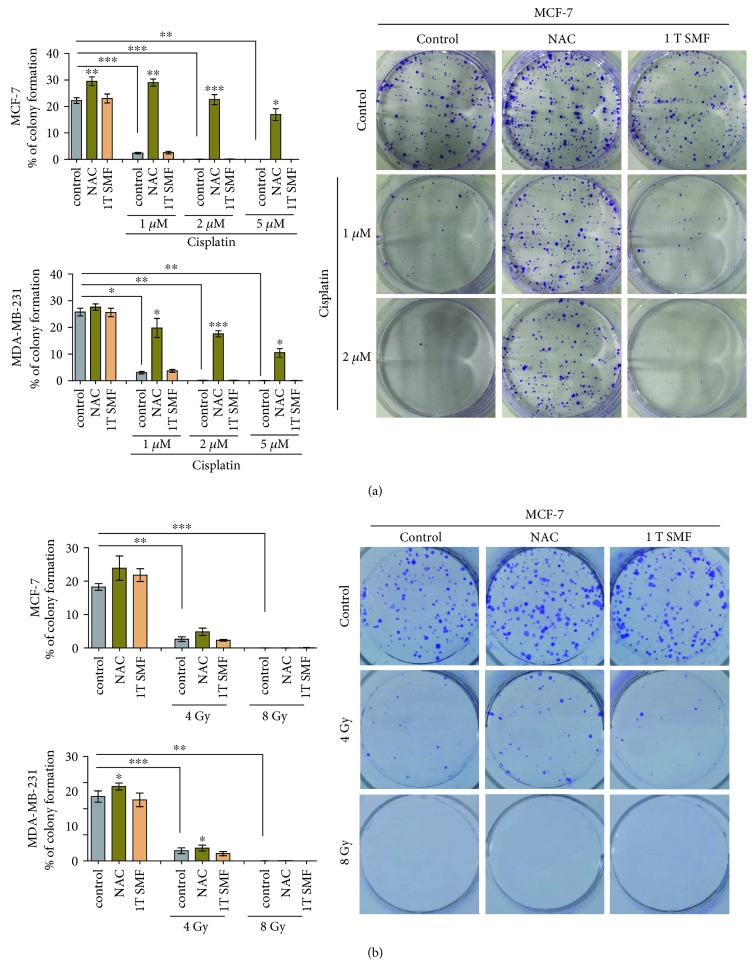
The effects of cisplatin, but not X-ray, on colony formation of MDA-MB-231 and MCF-7 cells are ROS dependent. The colony formation efficiency of MDA-MB-231 and MCF-7 cells with or without cisplatin, X-ray, NAC, and 1 T static magnetic field was examined. Comparisons were made between the experimental group and the control group using a Student's *t*-test. ^∗^ *p* < 0.05, ^∗∗^ *p* < 0.01, ^∗∗∗^ *p* < 0.001; ns: not significant.

Next, we did a clonogenic survival assay with radiation, cisplatin, NAC, and 1 T SMF, which is the best way of measuring the reproductive viability of cells after radiation and chemodrug treatments and the best way to show if their effects are rescued with NAC and/or SMF. It is obvious that cisplatin-induced colony formation reduction can be dramatically reversed by NAC treatment (Figure 6(a)). In contrast, NAC does not have obvious effect on X-ray-induced colony formation reduction (Figure 6(b)). These results indicate that the effects of cisplatin, but not X-ray, on colony formation of MDA-MB-231 and MCF-7 breast cancer cells are ROS dependent.

**Figure 7:**
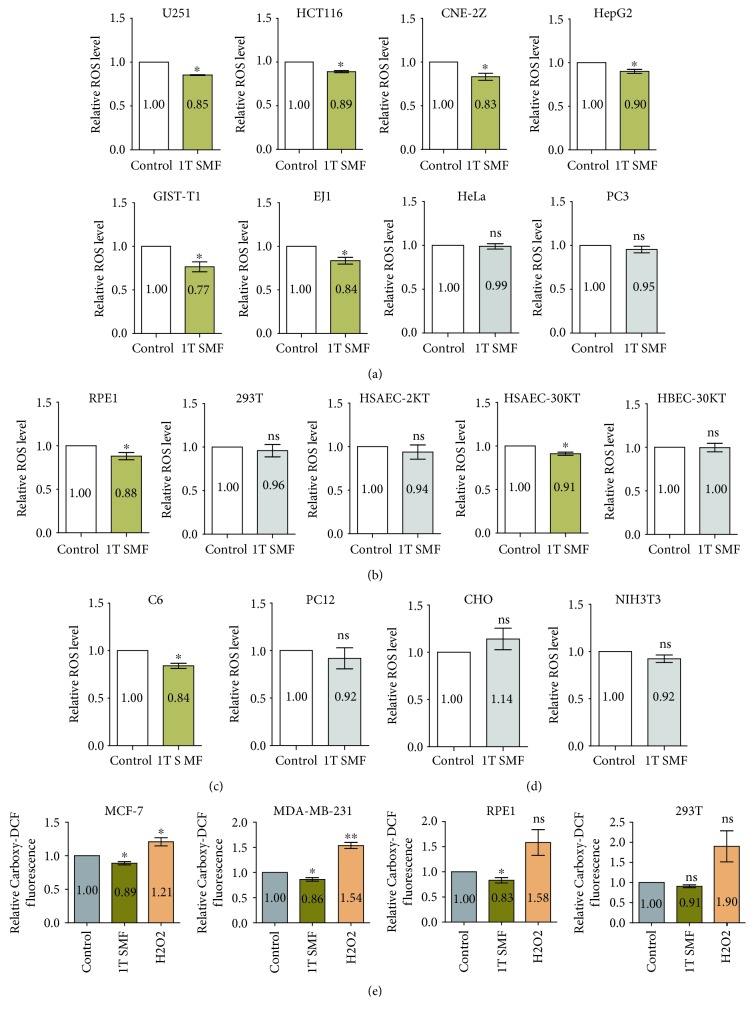
1 T static magnetic field decreases the intracellular ROS level in multiple cell types. Cells were plated at 4 × 10^5^/ml and treated with 1 T SMF for one day before they were harvested and measured for ROS levels. (a) Eight human cancer cell lines, (b) five human noncancer cell lines, (c) two rodent cancer cell lines and (d) two rodent noncancer cell lines were measured using the DCFH-DA probe. (e) Four cell lines were measured using Carboxy-H_2_DCFDA probe. Comparisons were made between the experimental group and the control group using a Student's *t*-test. ^∗^ *p* < 0.05, ^∗∗^ *p* < 0.01; ns: not significant. Brown color illustrates the ones that have statistical significance (*p* < 0.05).

We noticed that NAC itself could cause cell cycle alteration and colony formation increase while 1 T SMF did not (Figures 5 and 6). This is probably due to the much weaker ROS reduction effect of 1 T SMF compared to NAC in these two breast cancer cell lines (Figure 3). We then further examined the effect of 1 T SMF on ROS levels of 17 other cell lines, including 8 human cancer cell lines (Figure 7(a)), 5 human noncancer cell lines (Figure 7(b)), 2 rodent cancer cell lines (Figure 7(c)), and 2 rodent noncancer cell lines (Figure 7(d)). To further validate the effect of 1 T SMF on ROS level in two breast cancer cell lines (MDA-MB-231 and MCF-7) and two noncancer cell lines (293T and RPE1), we also used carboxy-H_2_DCFDA probe, which is an oxidation-insensitive ROS indicator (Figure 7(e)). It is interesting that 1 T SMF generally has a ROS reduction effect; more specifically, it decreased the ROS levels by ~10-20% in 11 out of 19 cell lines we tested in this study with statistical significance (Figure 7).

## 4. Discussion

Chemotherapy drugs and radiotherapy-induced cancer cell apoptosis frequently involves ROS overproduction [47], but the relationship between ROS and cancer still remains partially understood. The goal of the present study was to elucidate the role of ROS in response to X-ray exposure and cisplatin treatment and to determine the contribution of ROS in determining the anticancer efficacy of X-ray and chemodrug in breast cancer MDA-MB-231 and MCF-7 cells. This is helpful for designing therapeutic strategies to improve radiotherapy and chemotherapy efficacy in breast cancers and to reduce their toxicities.

One limitation of our study is that we did not provide detailed information about the specific types of ROS, such as peroxides, superoxide, or hydroxyl radical, because DCFH-DA and NAC are both general reagents for multiple types of ROS. DCFH-DA is a general ROS indicator that could react with various ROS and oxidizing species, such as hydrogen peroxide, superoxide, and peroxyl radical [48], and is also frequently used as a H_2_O_2_ indicator due to its higher reactivity to H_2_O_2_ [49, 50]. But we further used an oxidation-insensitive probe carboxy-H_2_DCFDA to confirm the effect of 1 T SMF on the ROS levels in two breast cancer cell lines and two noncancer cell lines. The fact that NAC, the scavenger for multiple types of ROS [42, 43], does not reverse the cell number reducing and cell death promoting effects of X-rays indicates that in general, ROS are not required for the anticancer efficacy of X-rays for MCF-7 and MDA-MB-231 breast cancer cells but are indispensable for cisplatin-induced anticancer effect.

Understanding the exact role of ROS in each physiological and pathological condition is essential. ROS are not only indispensable players in multiple cellular processes, such as cell proliferation, autophagy, and migration, they also play critical roles in cell death induction. However, it should be noted that ROS could also inhibit apoptosis [51–53], which may be used to explain the further cell number reduction caused by combining NAC and X-ray in MCF-7 cells in Figure 4(b). Further analysis of detailed ROS subtypes could provide more information on this aspect.

We have used four different X-ray doses (4-10 Gy) and two different breast cancer types (MDA-MB-231 and MCF-7) and found that the cell number reduction of X-rays is not dependent on ROS. However, we do not exclude the possibility that other doses and/or other cell types may be different. The spatiotemporal fluctuations of ROS rely on cellular redox systems, including NADH and NADPH, the concentrations of reduced glutathione (GSH)/oxidized glutathione (GSSG), and their transform relevant enzymes, such as glutathionylation and glutaredoxin [54–56]. There are many studies showing time-dependent ROS changes caused by radiation. For example, although the intracellular ROS level was dramatically increased at 24 h after 4 Gy X-ray irradiation, it also declined at 48-72 h or even longer timepoints in several cell lines, including MCF-7 [57], human leukaemia K562 and HL60 cells [10], and hematopoietic stem/progenitor HSPCs cells [58]. However, it should be noted that even after ROS levels decline, they are still much higher than control groups. Moreover, there are also several studies showing that ROS sustained the same level after 24 h to 48 h exposure to SMF or other types of magnetic fields [59–61].

Our results revealed the potential antioxidant effects of 1 T SMF. In 19 cell lines we tested, it reduced the ROS levels in 11 of them by around 10-20% with statistical significance (*p* < 0.05). It has been reported that ROS levels could be affected by various electromagnetic fields, which vary among electromagnetic fields with different parameters, as well as biological samples examined [40]. Here, we used neodymium N38 permanent magnets with the north pole beneath the cells so that the magnetic field intensity at the cells is ~1 T. This condition has been shown to be able to inhibit multiple cancer cell proliferation in multiple cell types [34, 35, 62]. It is interesting that we also found 1 T SMF reduced ROS levels in more cancer cell lines (9 out of 12, ~83%) than in noncancer cell lines (2 out of 7, ~29%), which is probably due to the fact that cancer cell lines generally have higher ROS levels than noncancer cell lines or because cancer cells have a damaged ROS control system. It is possible that reducing the ROS levels in these cell lines reduced their proliferation rate. The effect of magnetic fields with other parameters, such as different magnetic field intensities, on various cell types will be further examined.

In conclusion, our study shows that 4-10 Gy X-rays reduce MDA-MB-231 and MCF-7 breast cancer cell number in an ROS-independent way. Decreased ROS levels prevented cisplatin from reducing breast cancer cell number and causing cell death but did not reduce that of X-rays, except for some moderate effects on X ray-induced cell cycle arrest. This indicates that antioxidant agents may be used to reduce ROS-related side effects of radiotherapy without sacrificing its anticancer efficacy in breast cancer patients. Moreover, we found that 1 T SMF could reduce ROS levels in multiple cell lines. The underlying mechanism of 1 T SMF likely involves the interaction between the magnetic field and mitochondria, which will need further investigations.

## Data Availability

The data used to support the findings of this study are included within the article.

## Supplementary Materials

Supplementary Materials Supplementary Figure 1: the 1 T static magnetic field (SMF) exposure. Supplementary Figure 2: the raw data of cell cycle analysis for Figure 5.
