# Supplementary material for: ROS Reduction Does Not Decrease the Anticancer Efficacy of X-Ray in Two Breast Cancer Cell Lines
Source: Oxid Med Cell Longev. 2019 Mar 14;2019:3782074. doi: 10.1155/2019/3782074 (PMC6437742; doi:10.1155/2019/3782074)
Supplement: Supplementary Materials — Supplementary Figure 1: the 1 T static magnetic field (SMF) exposure. Supplementary Figure 2: the raw data of cell cycle analysis for Figure 5. [file 3782074.f1.pdf]

Supplementary Figure 1

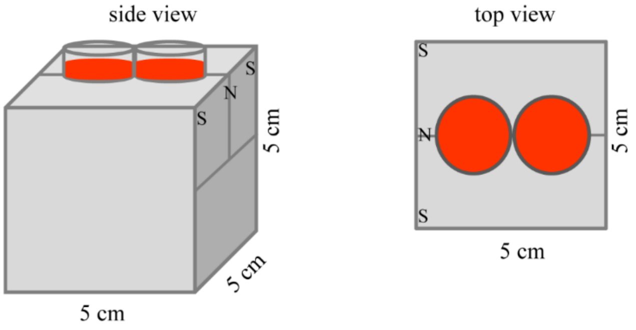

Supplementary figure 1. The 1T static magnetic field (SMF) exposure. The SMF was provided by placing the cell plate on the top center of a 5 cm × 5 cm × 5 cm neodymium permanent magnet (measured surface magnetic field intensity is 1.07 ± 0.037 T), with the North (N) pole down. The diameter of the cell culture plate in this study is 2 cm.

Supplementary Figure 2

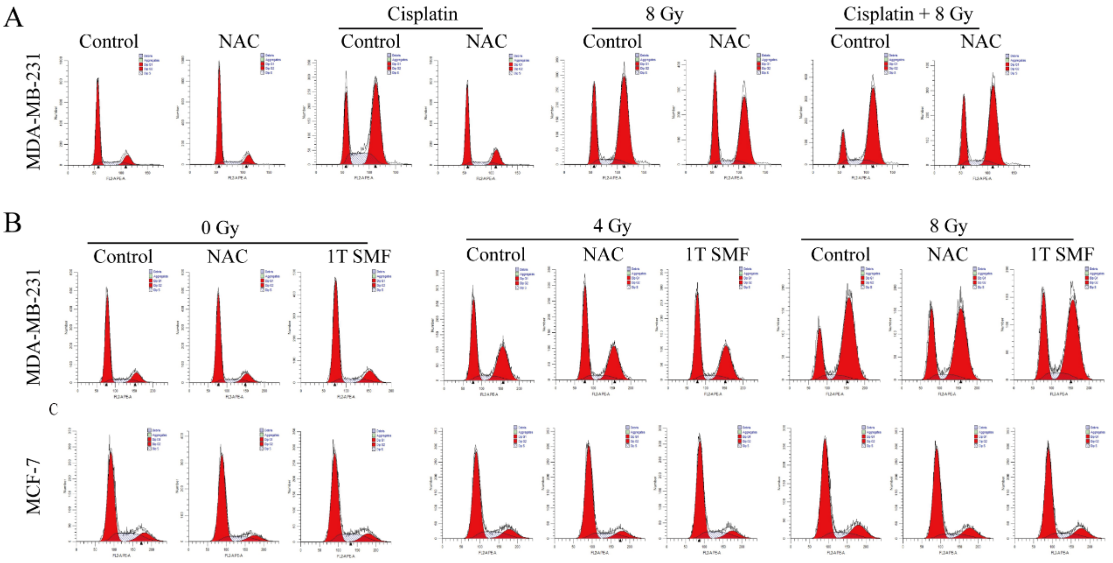

Supplementary Figure 2. The raw data of cell cycle analysis for figure 5.
